# Clinical significance of pleural fluid lactate dehydrogenase/adenosine deaminase ratio in the diagnosis of tuberculous pleural effusion

**DOI:** 10.1186/s12890-024-03055-0

**Published:** 2024-05-15

**Authors:** Tingting Zhao, Jianhua Zhang, Xiufeng Zhang, Cheng Wang

**Affiliations:** 1 Department of Respiratory and Critical Care Medicine, Shandong Public Health Clinical Center, Shandong University, Shandong, 250013 China; 2 Department of Thoracic Surgery, Shandong Public Health Clinical Center, Shandong University, Shandong, 250013 China

**Keywords:** Tuberculous pleural effusion, Parapneumonic pleural effusion, Malignant pleural effusion, Adenosine deaminase, Lactate dehydrogenase, Diagnostic value

## Abstract

**Background:**

Pleural fluid is one of the common complications of thoracic diseases, and tuberculous pleural effusion (TPE) is the most common cause of pleural effusion in TB-endemic areas and the most common type of exudative pleural effusion in China. In clinical practice, distinguishing TPE from pleural effusion caused by other reasons remains a relatively challenging issue. The objective of present study was to explore the clinical significance of the pleural fluid lactate dehydrogenase/adenosine deaminase ratio (pfLDH/pfADA) in the diagnosis of TPE.

**Methods:**

The clinical data of 618 patients with pleural effusion were retrospectively collected, and the patients were divided into 3 groups: the TPE group (412 patients), the parapneumonic pleural effusion (PPE) group (106 patients), and the malignant pleural effusion (MPE) group (100 patients). The differences in the ratios of pleural effusion-related and serology-related indicators were compared among the three groups, and receiver operating characteristic curves were drawn to analyze the sensitivity and specificity of the parameter ratios of different indicators for the diagnosis of TPE.

**Results:**

The median serum ADA level was higher in the TPE group (13 U/L) than in the PPE group (10 U/L, *P* < 0.01) and MPE group (10 U/L, *P* < 0.001). The median pfADA level in the TPE group was 41 (32, 52) U/L; it was lowest in the MPE group at 9 (7, 12) U/L and highest in the PPE group at 43 (23, 145) U/L. The pfLDH level in the PPE group was 2542 (1109, 6219) U/L, which was significantly higher than that in the TPE group 449 (293, 664) U/L. In the differential diagnosis between TPE and non-TPE, the AUC of pfLDH/pfADA for diagnosing TPE was the highest at 0.946 (0.925, 0.966), with an optimal cutoff value of 23.20, sensitivity of 93.9%, specificity of 87.0%, and Youden index of 0.809. In the differential diagnosis of TPE and PPE, the AUC of pfLDH/pfADA was the highest at 0.964 (0.939, 0.989), with an optimal cutoff value of 24.32, sensitivity of 94.6%, and specificity of 94.4%; this indicated significantly better diagnostic efficacy than that of the single index of pfLDH. In the differential diagnosis between TPE and MPE, the AUC of pfLDH/pfADA was 0.926 (0.896, 0.956), with a sensitivity of 93.4% and specificity of 80.0%; this was not significantly different from the diagnostic efficacy of pfADA.

**Conclusions:**

Compared with single biomarkers, pfLDH/pfADA has higher diagnostic value for TPE and can identify patients with TPE early, easily, and economically.

**Supplementary Information:**

The online version contains supplementary material available at 10.1186/s12890-024-03055-0.

## Background

Tuberculosis (TB) is an infectious disease caused by *Mycobacterium tuberculosis* and usually presents as a pulmonary infection. However, it may also invade extrapulmonary tissues or organs; such cases are called extrapulmonary TB. Tuberculous pleural effusion (TPE) is the second most common form of extrapulmonary TB, second only to lymph node involvement. It is the most common cause of pleural effusion in TB-endemic areas and the most common type of exudative pleural effusion in China [1, 2].

There is a certain difficulty in the clinical diagnosis of TPE, which relies on bacteriological and histopathological examinations including an acid-fast bacilli smear, rapid molecular tests, and culture of tuberculous bacteria, the latter of which is the gold standard. However, the positive rate of TB smear and culture in pleural effusion is low, and the time required for culture is too long to meet the needs of early clinical diagnosis [3]. The positive detection rate of biopsy by percutaneous pleural biopsy and thoracoscopy is high, but widespread application of this technique is difficult because of its invasiveness, complications such as hemopneumothorax, high cost, and need for highly skilled operators [4, 5], making the diagnosis of TPE somewhat challenging. Therefore, it is important to find easy, noninvasive, practical, and rapid auxiliary detection means and indicators. Several biomarkers have recently been applied to the diagnosis and differentiation of TPE in China and abroad, including adenosine deaminase (ADA), lactate dehydrogenase (LDH), C-reactive protein (CRP), and several inflammatory cytokines in serum and pleural effusion [6].

ADA, a nucleic acid-metabolizing enzyme related to the immune activity of cells, is present in almost all human tissues; however, it is most abundant in the lymphatic system, where its activity is closely related to immune function [7]. Multiple studies worldwide have shown that ADA has diagnostic significance for TPE because of its high sensitivity and specificity, and it is the most commonly used marker for diagnosing TPE in clinical practice. However, the cut-off value varies greatly among multiple studies, ranging from 10 to 70 U/L [8, 9]. Recent studies have shown that the ADA level may be higher in pleural effusion caused by empyema, malignant tumors, or autoimmune diseases such as rheumatoid arthritis and systemic lupus erythematosus because of the abundance of lymphocytes in these types of effusion [10–12]. In addition, because of the significant impact of individual and regional prevalence rates on negative results and fluctuations in advanced-age patients with TPE, the current ADA threshold is not applicable to these patients [13, 14]. Common types of pleural effusion that should be distinguished from TPE include parapneumonic pleural effusion (PPE) and malignant pleural effusion (MPE). The treatment of pleural effusion usually begins after determining its permeability and comparing LDH levels in pleural effusion and serum according to Light’s criteria [15]. LDH is a glycolytic enzyme and an important indicator reflecting the body’s inflammatory response. When tissue damage or pleural effusion occurs secondary to an underlying disease process, LDH in serum and surrounding tissues can enter the pleural effusion, leading to an increase in its level within the pleural effusion [16]. However, LDH in pleural effusion may become elevated in TPE, PPE, and MPE, especially in patients with PPE, which can be clinically classified into three subtypes based on the severity of the condition [17], namely uncomplicated PPE (UPPE), complicated PPE (CPPE), and empyema. As the complexity of PPE increases, LDH levels also rise, resulting in lower sensitivity of LDH in diagnosing different types of pleural effusions, thereby limiting the utility of LDH in identifying the cause of pleural effusions in individual patients [17–19]. Because of the current difficulty in diagnosing TPE with a single indicator as well as the limited research on the practical value of the difference in the LDH/ADA ratio between TPE and non-TPE (such as PPE and MPE) in clinical practice, this study was performed to investigate whether the combined detection of multiple indicators can improve the sensitivity and specificity of the diagnosis of TPE.

Considering the different mechanisms of elevated ADA and LDH and the possible relationship between pleural effusion and systemic inflammatory responses, this study explored the diagnostic performance of multiple parameters obtained from routine blood and pleural effusion detection, both individually and jointly. The study aim was to identify a simple parameter to distinguish TPE from pleural effusion of other causes, providing a theoretical basis for the early clinical diagnosis of TPE.

## Methods

### Study population

This study involved a retrospective collection of the clinical data of 618 patients who underwent first-ever treatment of pleural effusion at Shandong Thoracic Hospital from January 2017 to December 2019. They were divided into the TPE group (412 patients), PPE group (106 patients), and MPE group (100 patients).

### Inclusion criteria

#### TPE

The diagnostic criteria for TPE were (i) positive pleural effusion, pleural biopsy tissue, or sputum TB test and (ii) changes consistent with a tuberculous granuloma in the pleural biopsy specimen. The clinical diagnostic criteria were pleural effusion presenting as an exudate, an elevated ADA level (> 40 U/L), a strong positive tuberculin skin test, or a positive interferon-γ release test.

#### PPE

The diagnostic criteria for PPE were pleural effusion presenting as an exudate and the absence of specific manifestations such as TB and malignant tumors in the pleural biopsy specimen. Symptoms such as cough, expectoration, and fever were also present. Imaging findings included pulmonary parenchymal infection, an elevated peripheral white blood cell count, and absorption of the pleural effusion after antibiotic treatment. Uncomplicated parapneumonic effusion (UPPE) was defined when patients responded to antibiotic treatment alone; complicated parapneumonic effusion (CPPE) was defined when nonpurulent-appearing effusions required medical interventions such as drainage and other procedures; and empyema was defined when there was frank pus in the pleural space.

#### MPE

The diagnostic criterion for MPE was the presence of malignant tumor cells in pleural effusion or pleural tissue.

### Exclusion criteria

The exclusion criteria for this study were as follows: (i) other possible causes of pleural effusion including heart failure, hypoproteinemia, nephrotic syndrome, liver cirrhosis, and autoimmune diseases; (ii) a positive HIV test; (iii) treatment with immunosuppressive therapy; (iv) TB combined with an infection from other parts of the body; (v) recent anti-TB treatment for more than 1 week, anti-infection treatment for more than 3 days, or anti-tumor treatment; and (vi) no pleural effusion cell classification.

### Clinical data collection

The following patient data were collected: general clinical data, including age, sex, and disease course; characteristics of the pleural fluid [location of pleural fluid, pleural fluid protein (pfPRO) level, pleural fluid ADA (pfADA) level, pleural fluid LDH (pfLDH) level, and pleural fluid glucose (pfGLU) level]; and peripheral blood test results [white blood cell count, lymphocyte count, serum ADA level, serum LDH level, albumin level, CRP level, and erythrocyte sedimentation rate (ESR)].

### Statistical methods

SPSS 22.0 (IBM Corp., Armonk, NY, USA) was used for the statistical analysis. Quantitative data conforming to a normal distribution are expressed as mean ± standard deviation, and those with a non-normal distribution are expressed as median (25th, 75th quartile). Categorical variables are expressed as rate or percentage. Single-factor analysis of variance was used. When the variance was uniform, the Student–Newman–Keuls test was used for pairwise comparison among the three groups. When the variance was uneven, the non-parametric Kruskal–Wallis test was used. Pearson’s chi-square test or Fisher’s exact test was used to compare the differences in rates between the groups. The Bonferroni method was used to adjust the test level for pairwise comparisons between groups. A *P* value of < 0.05 was considered statistically significant. The sensitivity and specificity of various indicators used for the evaluation of TPE were calculated. Data with a *P*-value of < 0.001 in the comparison of laboratory parameter ratios between groups were included in the receiver operating characteristic curve (ROC) analysis to evaluate the diagnostic performance of each indicator. The area under the ROC curve was calculated, the optimal diagnostic threshold was analyzed and obtained, and the clinical values of different indicators for the diagnosis of the same disease were compared.

## Results

### Comparison of basic characteristics and laboratory indicators among patients in TPE, PPE, and MPE groups

The patients’ clinical and laboratory characteristics are shown in Table 1. The age at onset was lowest in the TPE group (38.74 ± 19.22 years) and highest in the MPE group (60.99 ± 13.04 years), with a statistically significant difference (*P* < 0.001). The white blood cell count was lowest in the TPE group (5.93 × 10^9^/L) and highest in the PPE group (11.74 × 10^9^/L); that in the MPE group showed a slight increase (*P* < 0.001). The median CRP level (120.00 mg/L) and ESR (74 mm/h) in the PPE group were significantly higher than those in the TPE group, and those in the MPE group were lowest among all three groups (*P* < 0.001). The serum albumin level was lowest in the PPE group (median, 32.6 g/L). The absolute value of serum lymphocytes was lowest in the TPE group (1.18 × 10^9^/L) and highest in the PPE group (1.51 × 10^9^/L).

**Table 1:** Comparison of clinical and laboratory test results in patients with different types of pleural effusion (TPE, PPE, and MPE)

	TPE (*n* = 412)	PPE (*n* = 106)	MPE (*n* = 100)	*P* value
**Age, years**	38.74 ± 19.22	53.66 ± 15.46^**^	60.99 ± 13.04^**^	< 0.001
**Sex, male**	299 (72.6)	88 (83.0) ^*^	53 (53.0) ^**^	< 0.001
**Serum WBC, ×10** ^**9**^ **/L**	5.93 (4.90, 7.40)	11.74 (8.25, 17.53) ^**^	6.74 (5.44, 9.19) ^**^	< 0.001
**Serum LYM, ×10** ^**9**^ **/L**	1.18 (0.91, 1.49)	1.51 (1.23, 1.90) ^**^	1.30 (0.98, 1.60)	< 0.001
**sADA, U/L**	13 (10, 17)	10 (7, 15) ^**^	8 (7, 10) ^**^	< 0.001
**sLDH, U/L**	179 (156, 207)	183 (157, 219)	190 (164, 246) ^*^	0.065
**ALB, g/L**	37.5 (35.0, 40.1)	32.6 (28.2, 36.1) ^**^	37.8 (34.9, 40.7)	< 0.001
**ESR, mm/h**	42 (28, 61)	74 (50, 88) ^**^	28 (15, 49) ^**^	< 0.001
**CRP, mg/L**	40.90 (18.25, 75.45)	120.00 (68.00, 172.00) ^**^	18.70 (5.95, 40.90) ^**^	< 0.001
**pfADA, U/L**	41 (32, 52)	43 (23, 145) ^*^	9 (7, 12) ^**^	< 0.001
**pfLDH, U/L**	449 (293, 664)	2542 (1109, 6219) ^**^	357 (234, 543) ^*^	< 0.001
**pfPRO, g/L**	50.1 (46.0, 53.8)	47.9 (40.2, 52.8) ^**^	42.7 (37.0, 48.8) ^**^	< 0.001
**pfGLU, mmol/L**	4.62 (3.54, 5.67)	1.23 (0.32, 4.49) ^**^	5.86 (4.40, 7.38) ^**^	< 0.001

Data are presented as mean ± standard deviation, n (%), or median (25th, 75th percentile)

*TPE* Tuberculous pleural effusion, *PPE* Parapneumonic pleural effusion, *MPE* Malignant pleural effusion, *WBC* White blood cell; LYM, lymphocyte, *sADA* Serum adenosine deaminase, *sLDH* Serum lactate dehydrogenase, *ALB* Albumin, *ESR* Erythrocyte sedimentation rate, *CRP* C-reactive protein, *pfADA* Pleural effusion adenosine deaminase, *pfLDH* Pleural effusion lactate dehydrogenase, *pfPRO* Pleural effusion protein, *pfGLU* Pleural effusion glucose

*P < 0.05, **P < 0.01 compared with TPE group

The serum ADA level in the TPE group (13 U/L) was significantly higher than that in the PPE group (10 U/L) and MPE group (10 U/L). There was no significant difference in the serum LDH level among the groups. The median pfADA level in the TPE group was 41 (32, 52) U/L; the lowest was in the MPE group at 9 [7, 12] U/L, and the highest was in the PPE group at 43 (23, 145) U/L. The differences between the three groups were statistically significant (*P* < 0.001). The pfLDH level in the PPE group was 2542 (1109, 6219) U/L, which was significantly higher than that in the TPE group [449 (293, 664) U/L] (*P* < 0.001). The median pfPRO level in the TPE group was 50.1 g/L, which was slightly higher than that in the other two groups. The median pfGLU level was 4.62 mmol/L, which was slightly higher than that in the MPE group but significantly lower than that in the PPE group (1.23 mmol/L) (*P* < 0.001).

### Comparison of laboratory parameter ratios between TPE and non-TPE patients

The ratios of various laboratory parameters were calculated. Table 2 shows a summary of 14 parameter ratios with a *P* value of < 0.001 between the TPE group and the non-TPE group. Among these parameters, pfLDH and pfADA were involved in eight (57.14%) and seven (50.00%) ratio parameters, respectively. The *P* values for serum ALB/pfADA, pfLDH/pfADA, and pfLDH/pfGLU between the TPE, PPE, and MPE groups were all < 0.001. Subsequently, this study analysed the levels of pfLDH, pfADA, and pfLDH/pfADA ratio in TPE and three subgroups of PPE (UPPE, CPPE, and empyema) patients. The results also showed that the pleural fluid LDH/ADA levels in TPE patients were significantly lower than those in the subgroups of PPE (UPPE, CPPE, or empyema) (*P* < 0.001), as shown in Supplementary Material Table 1.

**Table 2:** Comparison of laboratory parameter ratios between TPE patients and non-TPE patients

	TPE (*n* = 412)	no-TPE (*n* = 206)		
		All no-TPE (*n* = 206)	PPE (*n* = 106)	MPE (*n* = 100)
**Age, years**	38.74 ± 19.22	57.26 ± 14.74	53.66 ± 15.46^**^	60.99 ± 13.04^**^
**Sex, male**	299 (72.6)	141 (38.4)	88 (83.0)	53 (53.0)
**CRP/pfADA**	0.65 (0.06, 1.51)	0.98 (0.25, 4.69) ^**^	1.41 (0.50, 5.43) ^**^	0.85 (0.00, 3.61)
**ESR/pfADA**	1.00 (0.61, 1.61)	1.95 (0.56, 4.14) ^**^	0.92 (0.32, 2.79)	2.73 (1.00, 4.91) ^**^
**ALB/pfADA**	0.90 (0.69, 1.17)	1.85 (0.36, 4.29) ^**^	0.58 (0.18, 1.38)^**^	4.12 (2.71, 5.57)^**^
**sLDH/pfADA**	4.16 (2.95, 5.72)	7.54 (1.35, 20.22)^**^	3.36 (1.20, 7.22)^**^	18.79 (12.14, 26.77)^**^
**pfADA/sADA**	3.11 (2.13, 4.08)	1.63 (0.93, 4.10)^**^	3.63 (1.59, 12.83)^*^	1.11 (0.84, 1.67)^**^
**pfLDH/CRP**	10.88 (6.29, 25.14)	28.05 (9.39, 88.21)^**^	31.48 (9.08, 121.30)^**^	18.19 (9.53, 68.93)^**^
**pfLDH/ESR**	11.44 (6.75, 17.94)	21.69 (10.55, 72.70)^**^	49.52 (18.14, 262.24)^**^	12.19 (7.14, 26.71)
**pfLDH/sADA**	34.40 (20.14, 52.94)	74.86 (29.32, 229.00)^**^	209.14 (49.69, 762.08)^**^	46.36 (26.00, 77.00)^**^
**pfLDH/ALB**	12.08 (7.87, 17.60)	26.59 (8.74, 104.24)^**^	12.30 (8.79, 17.45)^**^	8.74 (5.89, 13.98)^**^
**pfLDH/sLDH**	2.40 (1.61, 3.52)	5.28 (1.80, 19.57)^**^	18.31 (7.53, 38.04)^**^	1.80 (1.20, 2.66)^**^
**pfLDH/pfADA**	11.55 (8.61, 15.22)	49.17 (31.83, 68.08)^**^	55.45 (39.06, 75.37)^**^	40.31 (24.23, 60.00)^**^
**pfLDH/pfGLU**	100.16 (54.48, 178.52)	206.37 (53.55, 3830.49)^**^	3145.48 (278.18, 25122.22)^**^	55.34 (33.43, 125.05)^**^
**pfLDH/pfPRO**	9.12 (5.96, 13.46)	18.51 (8.33, 60.00)^**^	52.84 (23.96, 187.16)^**^	8.68 (4.93, 13.00)
**pfPRO/pfADA**	1.21 (0.99, 1.59)	2.49 (0.96, 4.68)^**^	1.04 (0.30, 2.13)^*^	4.68 (3.49, 6.50)^**^

Data are presented as mean ± standard deviation, n (%), or median (25th, 75th percentile)

*TPE* Tuberculous pleural effusion, *PPE* Parapneumonic pleural effusion, *MPE* Malignant pleural effusion, *pfADA* Pleural effusion adenosine deaminase, *sADA* Serum adenosine deaminase, *pfLDH* Pleural effusion lactate dehydrogenase, *sLDH* Serum lactate dehydrogenase, *ALB* Albumin, *ESR* Erythrocyte sedimentation rate, *CRP* C-reactive protein, *pfPRO* Pleural effusion protein, *pfGLU* Pleural effusion glucose, *sGLU* Serum glucose

*P < 0.05, **P < 0.01 compared with TPE group

### Diagnostic value of laboratory indicators and ratios in distinguishing TPE from non-TPE

**Fig. 1:**
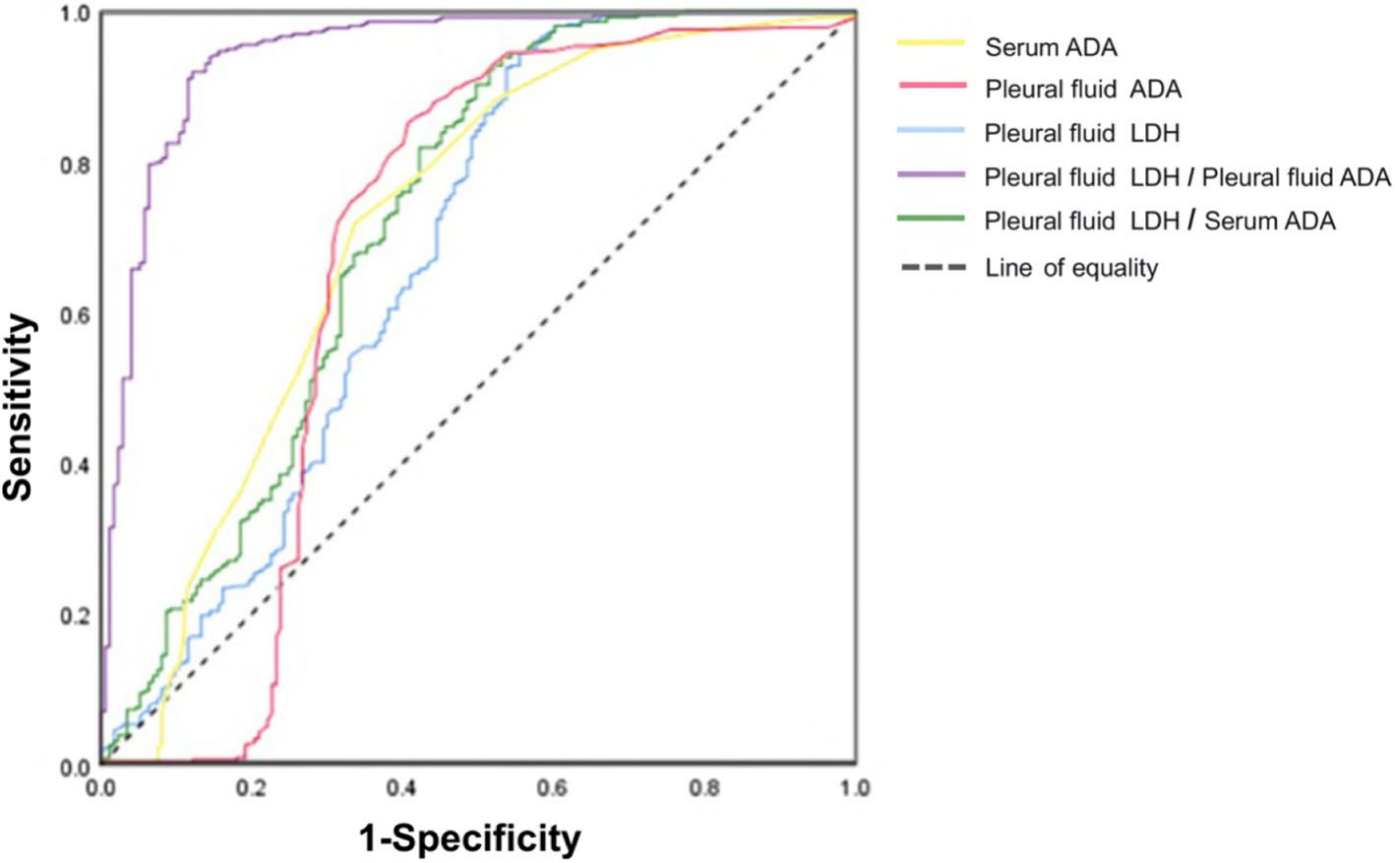
Receiver operating characteristic analysis of ADA and other significant indicators and their ratios to distinguish TPE from non-TPE. TPE, tuberculous pleural effusion; ADA, adenosine deaminase; LDH, lactate dehydrogenase

Figure 1 shows the results of the ROC analysis of the laboratory indicators and ratios used to distinguish TPE and non-TPE. The parameters with the highest area under the curve (AUC) were serum ADA, pfADA, pfLDH, pfLDH/pfADA, and pfLDH/serum ADA. Table 3 shows the diagnostic value of the laboratory indicators and ratios for distinguishing TPE from PPE. Among the single indicators, the AUC of serum ADA in the diagnosis of TPE was the highest at 0.713 (0.662, 0.765), with an optimal cut-off value of 10.5 U/L, sensitivity of 71.9%, specificity of only 66.5%, and Youden index of only 0.384. Among the parameter ratios, the AUC of pfLDH/pfADA for diagnosing TPE was the highest at 0.946 (0.925,0.966), with an optimal cutoff value of 23.20, sensitivity of 93.9%, specificity of 87.0%, and Youden index of 0.809. The differences in the AUCs of pfLDH/pfADA, pfLDH/serum ADA, and serum ADA were statistically significant (*P* < 0.001). Therefore, pfLDH/pfADA had the highest diagnostic value in distinguishing between TPE and non-TPE.

**Table 3:** Diagnostic value of laboratory indicators and ratios in distinguishing TPE from non-TPE

	AUC (95% CI)	*P* value	Cut-off	Sensitivity (%)	Specificity (%)	Youden index
**sADA**	0.713 (0.662–0.765)	0.026	> 10.5	71.9	66.5	0.384
**pfADA**	0.682 (0.626–0.737)	0.028	> 24.5	85.9	58.9	0.448
**pfLDH**	0.680 (0.630–0.731)	0.026	< 1032	94.2	45.4	0.396
**pfLDH/pfADA**	0.946 (0.925–0.966)	< 0.001	< 23.20	93.9	87.0	0.809
**pfLDH/sADA**	0.719 (0.667–0.771)	< 0.001	< 79.03	92.2	48.6	0.408

*TPE* Tuberculous pleural effusion, *AUC* Area under the curve, *CI* Confidence interval, *sADA* Serum adenosine deaminase, *pfADA* Pleural effusion adenosine deaminase, *pfLDH* Pleural effusion lactate dehydrogena

### Diagnostic value of laboratory indicators and ratios in distinguishing TPE from PPE and MPE

**Fig. 2:**
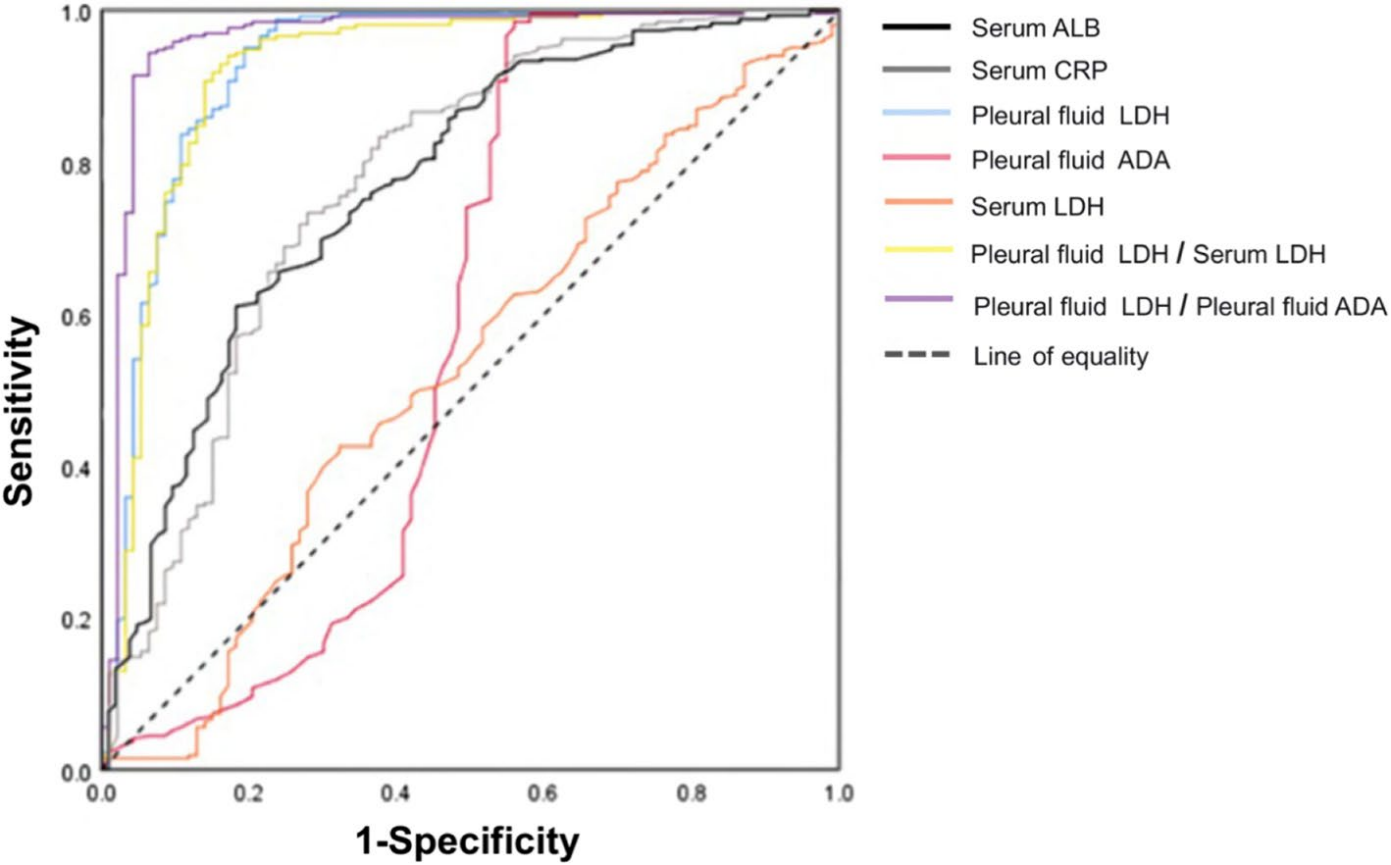
Receiver operating characteristic analysis of ALB and other significant indicators and their ratios to distinguish TPE from PPE. TPE, tuberculous pleural effusion; PPE, parapneumonic pleural effusion; ALB, albumin; CRP, C-reactive protein; ADA, adenosine deaminase; LDH, lactate dehydrogenase

**Fig. 3:**
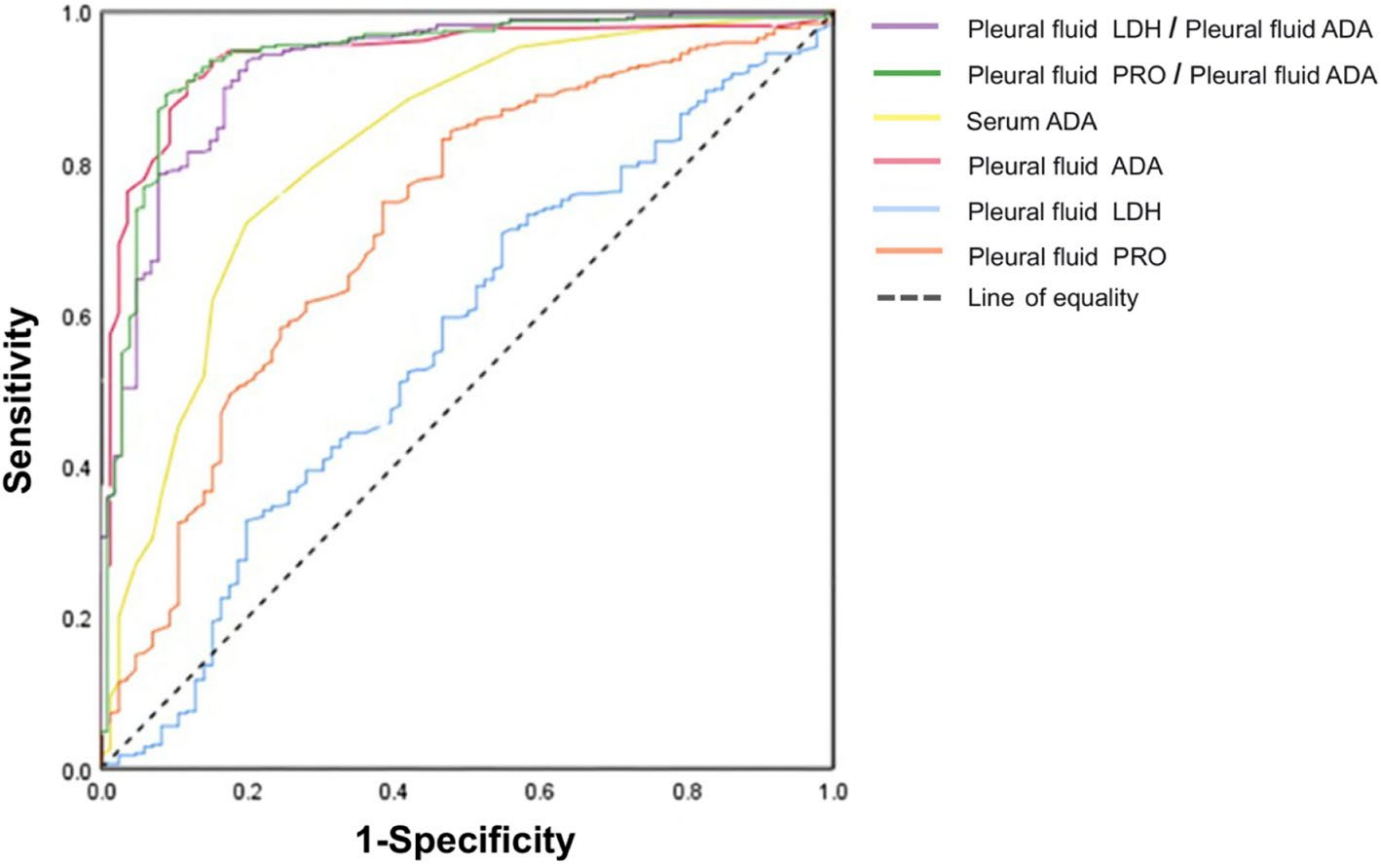
Receiver operating characteristic analysis of ADA and other significant indicators and their ratios to distinguish TPE from PPE. TPE, tuberculous pleural effusion; PPE, parapneumonic pleural effusion; ADA, adenosine deaminase; LDH, lactate dehydrogenase; PRO, protein

Figures 2 and 3 show the results of the ROC analysis of the laboratory indicators and ratios used to identify TPE, PPE, and MPE. In the differentiation of TPE and MPE (Table 4), the AUC of using pfLDH to distinguish TPE and PPE was the highest at 0.918 (0.882, 0.954), with an optimal cutoff value of 1064.5 U/L, sensitivity of 94.4%, specificity of 77.6%, and Youden index of 0.720. Among the parameter ratios, the AUC of pfLDH/pfADA was the highest at 0.964 (0.939, 0.989), with an optimal cut-off value of 24.32, sensitivity of 94.6%, and specificity of 94.4%. The AUC differences of pfLDH/pfADA with both pfLDH and pfADA are statistically significant (*P* = 0.019, *P* < 0.001). Therefore, it is recommended to use the pfLDH/pfADA ratio, which has higher diagnostic value, for the differential diagnosis of TPE and PPE.

**Table 4:** Diagnostic value of laboratory indicators and ratios in distinguishing TPE from PPE

	AUC (95% CI)	*P* value	Cut-off	Sensitivity (%)	Specificity (%)	Youden index
**pfLDH**	0.918 (0.882–0.954)	< 0.001	< 1064.5	94.4	77.6	0.720
**CRP**	0.778 (0.720–0.836)	< 0.001	< 94.85	84.3	62.6	0.469
**ALB**	0.770 (0.717–0.822)	< 0.001	> 36.65	61.0	81.7	0.427
**pfADA**	0.564 (0.485–0.643)	0.040	< 71	94.9	42.1	0.370
**sLDH**	0.520 (0.452–0.587)	0.549	< 173.5	44.0	65.3	0.093
**pfLDH/pfADA**	0.964 (0.939–0.989)	< 0.001	< 24.32	94.6	94.4	0.890
**pfLDH/sLDH**	0.929 (0.892–0.965)	< 0.001	< 5.41	92.6	85.1	0.777

*TPE* Tuberculous pleural effusion, *PPE* Parapneumonic pleural effusion, *AUC* Area under the curve, *CI* Confidence interval, *pfLDH* Pleural effusion lactate dehydrogenase, *CRP* C-reactive protein, *ALB* Albumin, *pfADA* Pleural effusion adenosine deaminase, *sLDH* Serum lactate dehydrogenase

In the differentiation between TPE and MPE (Table 5), the AUC of pfADA was 0.945 (0.922, 0.968), the sensitivity was 90.8%, and the specificity was 90.0%. The AUC of pfLDH/pfADA was 0.926 (0.896, 0.956), with a sensitivity of 93.4% and a specificity of 80.0%. There was no significant difference between them (*P* = 0.086). Therefore, in addition to pfADA, pfLDH/pfADA also has certain value in the differential diagnosis of TPE and MPE.

**Table 5:** Diagnostic value of laboratory indicators and ratios in distinguishing TPE from MPE

	AUC (95% CI)	*P* value	Cut-off	Sensitivity (%)	Specificity (%)	Youden index
**pfADA**	0.945 (0.922–0.968)	< 0.001	> 18.50	90.8	90.0	0.808
**sADA**	0.819 (0.766–0.872)	< 0.001	> 10.50	72.0	80.2	0.522
**pfPRO**	0.740 (0.684–0.795)	< 0.001	> 43.45	84.7	54.0	0.384
**pfLDH**	0.574 (0.509–0.639)	0.022	> 318.5	70.9	46.0	0.169
**pfPRO/pfADA**	0.937 (0.907–0.967)	< 0.001	< 2.13	89.1	91.0	0.801
**pfLDH/pfADA**	0.926 (0.896–0.956)	< 0.001	< 22.92	93.4	80.0	0.734

*TPE* Tuberculous pleural effusion, *MPE* Malignant pleural effusion, *AUC* Area under the curve, *CI* Confidence interval, *pfADA* Pleural effusion adenosine deaminase, *sADA* Serum adenosine deaminase, *pfPRO* Pleural effusion protein, *pfLDH* Pleural effusion lactate dehydrogenase

## Discussion

TPE is a common type of extrapulmonary TB and the most common type of exudative pleural effusion in China [17]. In addition to TB, malignant tumors and infections can also lead to pleural effusion. Their early clinical manifestations have no specific imaging findings, but their treatment plans, prognoses, and outcomes are quite different. Correct differentiation of TPE from pleural effusion of other causes is of great significance to the selection of clinical treatment methods. The diagnosis of TPE mainly depends on bacteriologic and histopathologic examination, but diagnosis remains difficult because of the low positive rate of *M. tuberculosis* culture, the long time required for culture, the invasive nature of tissue biopsy, and other factors [3–5]. This study explored the diagnostic value of individual and combined detection of multiple indicators obtained from blood and pleural effusion for TPE, with the aim of identifying a simple parameter to distinguish TPE from pleural effusion of other causes. The results showed that pfLDH/pfADA has good sensitivity and specificity in distinguishing between TPE and non-TPE (including PPE and MPE) and has high clinical diagnostic value, providing a theoretical basis for early clinical diagnosis of TPE.

The results of this study showed that there were differences in age and sex ratio among the TPE, PPE and MPE groups. Patients with TPE were relatively young, with a mean age of 38.74 years at the time of diagnosis; this was much lower than the mean age of patients with MPE (60.99 years) and PPE (53.99 years). Most patients with TPE (72.6%) and PPE (83.0%) were male. Among patients with MPE, the male-to-female ratio was close to 1, which is consistent with previous studies. These findings indicate that TPE is more common in young and middle-aged men, whereas MPE is more common in patients of advanced age [11, 20].

Biological markers, particularly pfADA, are crucial for TPE diagnosis in the clinical setting due to their convenience, speed, and cost-effectiveness. ADA contains two isoenzymes, ADA1 and ADA2, of which ADA2, predominant in monocytes and macrophages, is more specific for TPE detection than total ADA [21, 22]. However, due to non-standardized and costly ADA2 assays, total ADA levels in pleural effusion remain the primary marker in the clinical setting.The currently accepted ADA cut-off value is 40 U/L [9], but the threshold value ranges from 17.5 to 77 U/L among different studies and decrease with age [8, 9, 23–27]. LDH is a widely distributed hydrogenated reductase that plays an important role in sugar metabolism. pfLDH is mostly used to identify the nature of an effusion as an exudate, which is considered when the pfLDH/serum LDH ratio is < 0.6 or pfLDH is greater than two-thirds the upper limit of normal for serum LDH [15]. Pleural effusions from patients with empyema contain significantly higher levels of LDH, second to MPE, but only slightly higher levels than serum in patients with TPE [28, 29]. However, use of pfLDH is still limited because of its low sensitivity [19]. Therefore, distinguishing TPE from non-TPE by pfADA and pfLDH levels remains challenging. In recent years, molecular markers like interleukins 27, 31, and 33 show promise but are costly and impractical in TB-heavy developing regions. This study aims to identify a practical parameter for early TPE diagnosis using current techniques [30–33]. The present study was performed with the aim of identifying a feasible parameter to assist the early diagnosis of TPE by current detection techniques.

In this study, the median pfADA level for TPE was 41 U/L, close to the accepted cut-off value and lower than the level of 43 U/L in patients with PPE. Previous studies have shown that the pfADA level in patients with PPE was not higher than that in patients with TPE, but the heavier infection and more complex conditions of the PPE patients in this hospital study should be considered [19, 29]. Clinically, PPE patients can be divided into different subtypes (UPPE, CPPE and empyema) according to the severity of the disease. As shown in this study, the pfADA level of UPPE patients was significantly lower than that of TPE patients, while the pfADA level of CPPE and empyema patients was significantly higher than that of TPE patients. In TPE, TB candisrupt lymphocyte-mediated cellular immunity, with ADA closely linked to T-cell proliferation, differentiation, and numbers. Lymphocytes exhibit enhanced proliferation and differentiation under TB antigen stimulation, leading to increased ADA levels. Conversely, in MPE, low ADA levels are observed due to suppressed immune function in cancer patients, resulting in reduced ADA activity in lymphocytes [34]. In this study, both serum ADA and pfADA were significantly higher in TPE patients than in patients with MPE, consistent with previous studies [35]. In patients of advanced age, false-negative results may occur with ADA, whereas misdiagnosis may occur with higher ADA levels when differentiating from bacterial pleural infections (particularly complex PPE and empyema) and lymphoma [36]. Alternatively, ADA levels may be higher in pleural effusions caused by autoimmune diseases such as rheumatoid arthritis and systemic lupus erythematosus [37]. Therefore, ADA cannot be used as a clinically independent diagnostic indicator for TPE.

LDH, crucial in sugar metabolism and prevalent in tumor tissues, is often elevated in MPE due to tumor cells favoring the LDH-driven anaerobic glycolytic pathway for energy production [38]. Higher CRP levels in patients with lung cancer than in healthy individuals have also been reported [39]. However, CRP elevation could not be attributed to cancer in the present study because patients with lung cancer may also have inflammation such as cancer-related lung infection. In MPE patients, CRP and ESR levels were raised but lower than in PPE and TPE cases. Moreover, pfLDH levels were notably higher in PPE patients. These findings align with prior research [19, 29]. However, some studies have found that there is no significant difference in pfLDH level between UPPE and TPE patients [17], so it is challenging to use pleural effusion LDH to completely distinguish TPE from PPE, especially different PPE subtypes. Therefore, utilizing parameters such as ADA, LDH, CRP, and ESR together could enhance the accuracy of distinguishing between TPE and pleural effusions of other origins. Saraya T et al. [40] first proposed the use of pfLDH/pfADA as a method with good sensitivity and specificity for distinguishing pleural effusions of different etiologies in clinical practice. The results of this study also showed that the ratio of pfLDH/pfADA in TPE patients was significantly lower than that in PPE patients, and this difference was also statistically significant among different PPE subtypes (UPPE, CPPE and empyema) and TPE patients (*P* < 0.001).

This study calculated the ratio of each laboratory parameter, summarized the parameter ratios showing a difference with a *P* value of < 0.001 between the groups, and carried out an ROC curve analysis. In distinguishing TPE from non-TPE patients, the serum ADA level had a higher AUC than the pfADA and pfLDH levels but a significantly lower AUC than the pfLDH/pfADA ratio. The optimal cut-off value of pfLDH/pfADA was 23.20, with a sensitivity of 93.9% and a specificity of 87.0%. Compared to previous studies, this indicator demonstrates better sensitivity and specificity in diagnosing TPE. Wang et al. [17] found a sensitivity of 93% and specificity of 62% for pfLDH/pfADA in distinguishing between TPE and PPE. Anar et al. [41] reported a sensitivity of 90% and specificity of only 59.85% for diagnosing TBP when the pfLDH/pfADA threshold was 28. Vieira et al. [42] suggested that the sensitivity and specificity for diagnosing TPE were both 79% when the pfLDH/pfADA threshold was 8.3. Saraya T et al. [40] found that a pfLDH to pfADA ratio greater than 15.5 and a pleural CEA level of less than 5ng/mL is indicative of PPE or empyema rather than TPE, MPE, or transudative pleural effusion (chronic renal failure/congestive heart failure), and this method has a sensitivity of 62.0%, a specificity of 91.0%. Additionally, in a study from a region with a high incidence of TB (> 5/100,000), the cut-off value of the pfLDH/pfADA ratio for distinguishing between TPE and non-TPE was 12.5 [43]. In a study from a region with a low incidence of TB, the optimal cut-off value of this ratio was also only 15 [44]. The sensitivity was lower than that in the present study, while the specificity was similar. This difference illustrates that in addition to the regional incidence, factors such as the condition of patients with TPE, the patients’ age, and the disease composition of the control group may also affect the optimal cut-off level and the evaluation of its diagnostic value when distinguishing TPE. What’s more, our study also suggests that the utility of the pf LDH/pfADA ratio is helpful even for patients with TPE who have low pfADA levels.

The study also investigated the diagnostic value of the parameter ratios when distinguishing TPE from PPE and MPE. Among individual biochemical parameters, pfLDH had the highest AUC in differentiating TPE from PPE, with an optimal cut-off of 1064.5 U/L. However, the pfLDH/pfADA ratio outperformed pfLDH alone, with a superior AUC of 24.32, offering high sensitivity (94.6%) and specificity (94.4%).Previous research [29] also highlighted significant differences in the pfLDH/pfADA ratio between TPE and PPE, where MPE or empyema/PPE patients showed notably higher ratios than TPE patients [28].Kim et al. [30] found that the pfADA/serum CRP ratio had higher diagnostic value than the pfADA/pfLDH ratio. It has also been shown that the pfLDH/pfADA ratio has an AUC of only 0.783 in distinguishing PPE/empyema from other pleural effusions, including PPE and MPE, indicating it may not surpass single parameters in diagnostic value [45]. These studies, performed in areas of different incidences and involving patients with different disease severities and control group compositions, have produced mixed evaluations of the diagnostic value of the pfLDH/pfADA ratio. Larger samples and multicenter experimental data are still needed to evaluate the actual diagnostic value of the pfLDH/pfADA in a population in a specific context in the clinical setting.

Lingyun Shao et al. and Maria Rosa Chitolina Schetinger et al. [20, 38] recommended the serum LDH/pfADA ratio for the differential diagnosis of TPE and MPE, and Ling Xu et al. [34] agreed that this ratio has high sensitivity and specificity, terming it the “carcinogenesis ratio.” In the present study however, serum LDH and pfLDH were not significantly different between MPE and TPE. This may have occurred because the study involved patients with TPE who were more severely ill than those treated in the outpatient setting. More severely ill patients may have higher LDH levels, thereby closing the gap in the LDH level between patients with MPE and TPE. In this study, the AUC of the serum LDH/pfADA ratio was significantly lower than that of the pfADA level and pfLDH/pfADA ratio, while there was no significant difference in the AUC between the latter two. Therefore, in addition to the pfADA level, the pfLDH/pfADA ratio can also be used to identify TPE and MPE.

## Conclusions

In summary, this study evaluated the diagnostic performance of biomarkers and novel parameters for routine testing. These new parameters are ratios composed of two biomarkers that can emphasize the difference between different types of pleural effusions. The pfLDH/pfADA ratio has a higher diagnostic value than the pfADA level for differentiating TPE from non-TPE (including PPE and MPE) and can be used to identify patients with TPE early, easily, and economically.

### Supplementary Information

Supplementary Material 1.

## Data Availability

The data used in the present study are available upon request to the corresponding author.

## Abbreviations

ADA: Adenosine deaminase
CRP: C-reactive protein
ESR: Erythrocyte sedimentation rate
LDH: Lactate dehydrogenase
MPE: Malignant pleural effusion
PPE: Parapneumonic pleural effusion
pfLDH: Pleural fluid lactate dehydrogenase
pfADA: Pleural fluid adenosine deaminase ratio
pfPRO: Pleural effusion protein
pfGLU: Pleural effusion glucose
ROC: Receiver operating characteristic curve
TB: Tuberculosis
TPE: Tuberculous pleural effusion
