# Supplementary material for: Clinical significance of pleural fluid lactate dehydrogenase/adenosine deaminase ratio in the diagnosis of tuberculous pleural effusion
Source: BMC Pulm Med. 2024 May 15;24:241. doi: 10.1186/s12890-024-03055-0 (PMC11097553; doi:10.1186/s12890-024-03055-0)
Supplement: Supplementary file 1 — Supplementary Material 1. [file 12890_2024_3055_MOESM1_ESM.docx]

Table 1. Comparison of pleural fluid LDH, ADA, and LDH/ADA ratio values between patients with TPE, UPPE, CPPE, and empyema

|  | TPE (412) | UPPE (22) | CPPE (76) | Empyema (8) | *P* value |
| --- | --- | --- | --- | --- | --- |
| pfLDH | 449(293,664) | 671.30 (462.25,817.250) **^a^** | 3186.500 (1788.50，6426.50) **^b^** | 26994.50 (3529.25, 32455.25) **^c^** | <0.001 |
| pfADA | 41 (32, 50) | 17.50 (12.25, 23.00) **^a^** | 73.00 (31.00, 149.00) **^b^** | 178. 50(64.50, 294.75) **^c^** | <0.001 |
| pfLDH/pfADA | 11.55(8.61,15.22) | 39.72 (29.82,47.21) **^a^** | 58.36 (41.38, 77.24) **^b^** | 115.84 (61.42, 138.13) **^c^** | <0.001 |

TPE, tuberculous pleural effusion; UPPE, uncomplicated parapneumonic pleural effusion;CPPE, complicated parapneumonic pleural effusion; MPE, malignant pleural effusion; pfADA, pleural effusion adenosine deaminase; pfLDH, pleural effusion lactate dehydrogenase; ^a^, the difference between UPPE and TPE group was statistically significant (*P*<0.05); ^b^, the difference between CPPE and TPE group was statistically significant (*P*<0.05); ^c^, the difference between empyema and TPE group was statistically significant (*P*<0.05).
